# Do the radiological changes seen at mid term follow up of stemless shoulder prosthesis affect outcome?

**DOI:** 10.1186/s12891-019-2870-z

**Published:** 2019-10-27

**Authors:** Mohamed Moursy, Milan Niks, Aditya S. Kadavkolan, Lars J. Lehmann

**Affiliations:** 1 Department of Orthopedics and Traumatology, Paracelsus Medical University, Salzburg, Austria; 2 Dept. of Traumatology, Emergency hospital Graz, Graz, Austria; 3 Consultant Arthroscopy, Sports Medicine & Shoulder surgery, Dr. LH Hiranandani Hospital, Powai, Mumbai, 400076 India; 4 Orthopedic & Trauma Centre, Medical Faculty Mannheim, Heidelberg University Mannheim, Mannheim, Germany

**Keywords:** Shoulder arthroplasty, Shoulder arthritis, Stemless arthroplasty, Eclipse prosthesis, Total shoulder arthroplasty

## Abstract

**Background:**

The Eclipse® (Eclipse® is a trademark of Arthrex, Naples, Florida) stemless shoulder prosthesis offers the surgeon the advantage of bone stock preservation and at the same time avoids the drawbacks of a resurfacing arthroplasty. Previous studies have shown radiographic changes on serial follow up of the Eclipse prosthesis. This study attempts to assess the significance of these radiographic changes and effect of cuff related pathology on the mid-term outcome of the Eclipse prosthesis.

**Methods:**

Between July 2005 and October 2008, 29 shoulders underwent shoulder arthroplasty with the Eclipse prosthesis; 23 shoulders, (seven women and 16 men) were available for the final follow up. The range of motion, Constant Score; age adjusted Constant Score, Subjective Shoulder Value and radiographs were assessed at serial follow-ups.

**Results:**

Significant improvements were seen in the Constant Score (78.9 ±20.1) compared to pre-operative score (32.9 ±5.2); also forward elevation, abduction and external rotation improved to 142.9 ± 36.6 °, 135.2 ± 40.5 ° and 49.8 ± 21.9 ° at 72 months (*p* < 0.001). Radiolucent lines and localised osteopenia, did not statistically impact on the clinical outcome. Partial tears of the supraspinatus and subscapularis had a negative impact on the Subjective Shoulder Value (*p* < 0.05) Partial or complete tears of the subscapularis led to worse Constant Score on follow up (*p* < 0.05).

**Conclusions:**

The presence of radiolucent lines or localised osteopenia does not influence the mid term clinical outcome. Pre -operative partial supraspinatus tears or tears of the subscapularis lead to an inferior outcome.

## Background

Modularity in current third and fourth generation prosthesis allows for restoration of shoulder biomechanics [1–3]. The last decade has seen an increase in the stemless implants so as to circumvent the complications associated with a stemmed prosthesis namely periprosthetic fractures and loosening. The average interval for loosening has been found to be 7.7 ± 4 years and for periprosthetic fractures the duration has been found to be 5.8 ± 4.7 years [4]. Intra operative perioperative fractures may occur due to forceful manipulation or a mistake in introduction of the stem in the diaphysis [5]. In a meta-analysis 27 of 414 complications in shoulder arthroplasty were due to loosening of the stem [6] To overcome the loss of bone stock and the stem associated complications humeral resurfacing arthroplasty was introduced [7]. The resurfacing offers accurate offset, as there is no stem; however angulation is prone to major errors [8]. Good results with shoulder resurfacing have been recorded in cases of rheumatoid arthritis, glenohumeral arthritis, in malunited humeral shaft fractures and in cases where there is an implant occupying the humeral shaft canal; difficulties are however seen in proximal humeral malunions and in patients with advanced collapse of the humeral head [5, 9, 10]. Also the procedure is technically demanding, requiring a circumferential capsulotomy for the glenoid exposure and humeral head protection during the glenoid preparation [7, 9].

Stemless implants offer the advantages of both resurfacing as well as the conventional stemmed prosthesis- a) accurate offset since there is no stem, b) correct head angulation and version c) easy extraction during revision [8] d) less compromise of bone stock and e) avoidance of diaphyseal stress shielding as more of the metaphyseal bone is loaded [11].

Previous studies have shown radiographic changes around the implant at sequential follow up, many of which were asymptomatic [12, 13].

Whereas rotator cuff disease limited to the supraspinatus with minimal or no retraction has not shown to affect the outcomes of anatomical total shoulder arthroplasty; worse outcomes are observed in individuals with fatty infiltration and degeneration of the infraspinatus and to a lesser extent the subscapularis [14]. Similar outcomes have been noted by Ianotti et.al who observed no significant effect of a repairable supraspinatus tear on the increase in American Shoulder Elbow Society scores for pain, function and patient satisfaction in individuals undergoing anatomical prosthetic replacement [15].

In the present study it was hypothesized that the radiographic changes were mainly due to bone adaptation / stress redistribution around the implant and would not influence the clinical outcome. As has been discussed previously, anatomic total shoulder arthroplasty in gives predictable outcomes in individuals with isolated supraspinatus tear; whether the same applies to a stemless shoulder arthroplasty has not been assessed. Hence one of the objectives of the current study was to evaluate the effect of rotator cuff tears on the clinical outcome of the stemless prosthesis.

## Methods

Between July 2005 and October 2008, 29 shoulders underwent shoulder arthroplasty with the Eclipse prosthesis performed by the senior author.

The patients were assessed clinically pre-operatively with the Constant –Murley score (CS) and the range of motion of the shoulder was evaluated. The clinical assessment was done by MM & MN; passive and active range of motion of the shoulder with regards to forward elevation, abduction and external rotation at side were assessed with a clinical goniometer. These measurements were again repeated at 6 weeks, 3 months, 6, 12, 24, 36 and 72 months. Additionally the age adjusted Constant Score and Subjective Shoulder Value (SSV) was used for measuring the outcome.

**Fig. 1:**
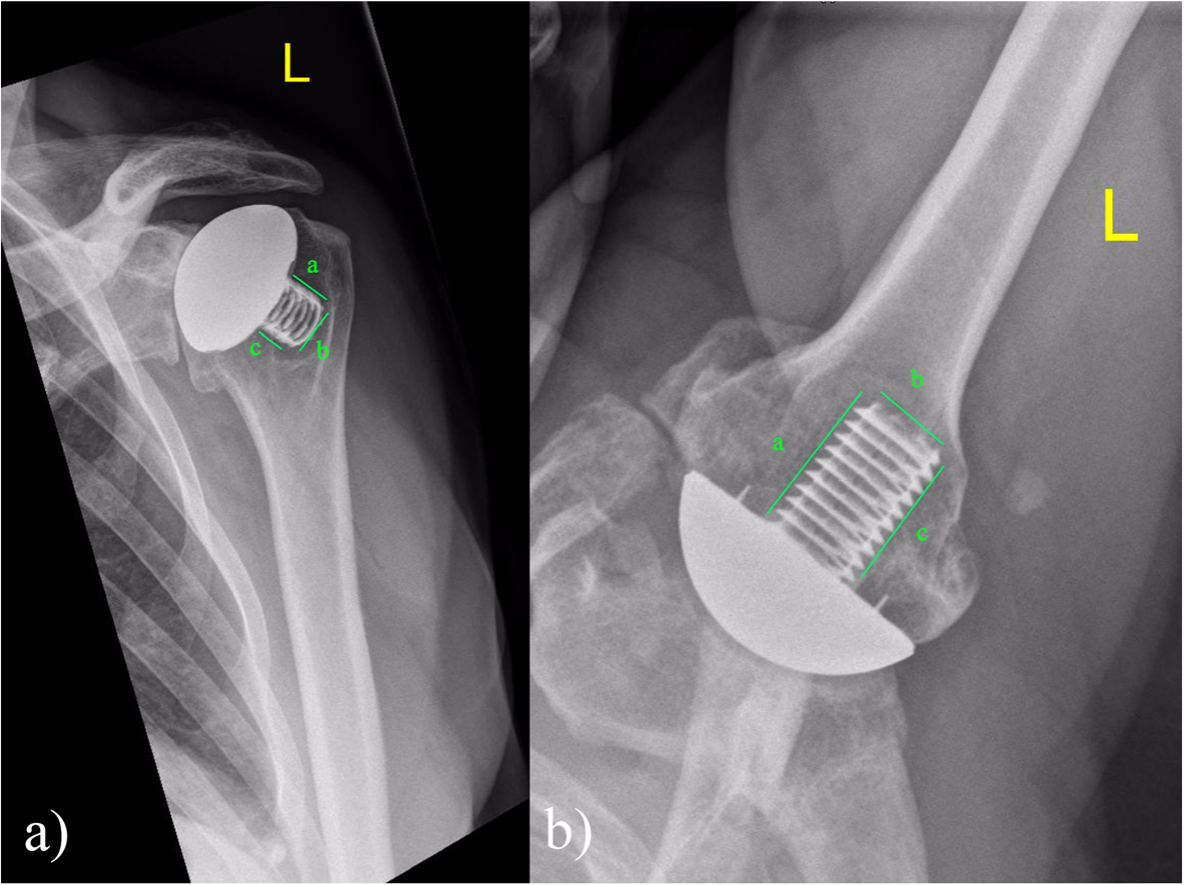
Assessment of the radiolucencies around the humeral component as described by Habermeyer, a,b,c represent the zones around the prosthesis. **a** AP View, **b** Axillary view

Radiographic evaluation was done with AP and axillary radiographs; radiolucencies and osteopenia were measured in three zones *a, b* and *c* around the implant base and coring screw as previously described by Habermeyer et.al in the Anteroposterior (AP) and axillary views of the shoulder [13] (Fig. 1). Stress shielding was assessed from tuberosity resorption, cortical thinning, and calcar osteolysis [16]. The radiographic observations were made by two observers trained and experienced in shoulder surgery MM & MN as per the system devised by Habermeyer [13]. The final conclusion of the radiological changes were based on inter observer agreement.

Pre operatively the rotator cuff was evaluated with the help of a 3 Tesla MRI with standard sequences. We assessed the MRI for presence rotator cuff tear, presence of fatty infiltration and retraction. Rotator cuff tears involving the supraspinatus , infraspinatus or the subscapularis were graded as nil (0), partial tear (1) or complete tear (2). Fatty infiltration was graded absent (1)(≤2 Goutallier) or present (≥2 Goutallier).

All the surgeries were performed under General Anaesthesia with the patient in the beach chair position. A deltopectoral approach from the coracoid extending inferior and laterally over the superior aspect of the axillary fold was used. The deltoid muscle was mobilized laterally and retracted. The lateral end of the subscapularis was identified and tag sutures with no. 5 fiberwire®^1^ were taken for later attachment of the muscle to the bone. A long head of biceps tenotomy was performed in all the cases. A capsular release till 6 ‘o clock position of the humerus was done and the head was dislocated anteriorly for resection. The arm was positioned at more than 90 ° external rotation and 30 ° extension and adduction for head resection.

The Eclipse prosthesis is an uncemented humeral head replacement prosthesis, which has a central coring screw for fixation to the humeral metaphysis. The implant is available in sequential sizes and progressive thickness to replicate the proximal humerus geometry. The coring screw is available in Small, Medium and Large. However there is no modularity between the humeral head and the screw. The cemented polythene glenoid component comes in two variants; a keeled and a pegged back both of which have reverse bars and fenestration to improve the fixation. The decision to implant the glenoid was based on the Walch classification [17]. Eccentric glenoids were implanted while A1 glenoids were left alone.

Following the removal of osteophytes the head was resected with a resection guide, the triunion size was determined and the metaphysis was drilled for the coring screw. The sterile and sized triunion was finally placed and impacted and the coring screw placed through the central hole following which the head was impacted. In shoulders that required the placement of the glenoid component, the glenoid preparation was done after the humeral head osteotomy. For glenoid component preparation the humerus was subluxed posteriorly after tenotomy of the superior aspect of the tendon of the pectoralis major. It is paramount to achieve complete visualization of the glenoid to achieve optimal component placement. A guide was introduced at the centre of the glenoid followed by reaming and placement of a polythene glenoid component.

The arm was left postoperatively in a Gilchrist immobilization bandage for three weeks during which passive range of motion was permitted after which active range of motion exercises and active mobilization was initiated.

Data were entered in Microsoft Excel and analysed using Stata Version 15.1 (© StataCorp, College Station, Texas, USA). The mean and standard deviations for the linear variables were calculated. The means between two groups were compared using the t-test. The analysis of variance to assess the difference in means in more than two groups was use. The Pearson’s correlation co-efficient was used to estimate the correlation between two linear variables; *p* < 0.05 was considered to be statistically significant.

## Results

No intra-operative complications were observed in any of the patients. subscapularis lengthening was performed in 17 patients. Two patients required a repair of the supraspinatus during the surgery. The mean operative time was 73± 15.2 min and the blood loss was 215 ±29.2ml.

**Fig. 2:**
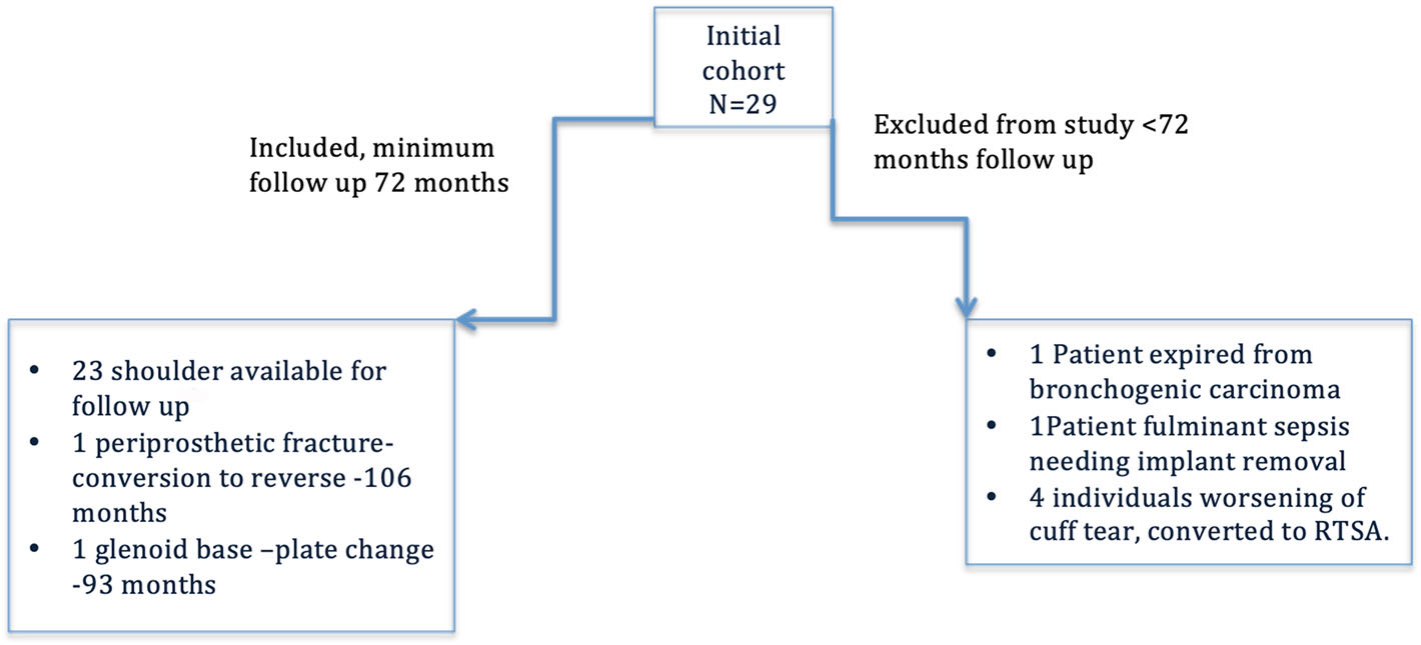
Flowchart depicting the inclusion/exclusion criteria in the study

Out of the 29 shoulders, 23 (seven women and 16 men) were available for the final follow up of which three were followed up through a self-assessment form. Only the individuals who had completed a minimum of 72 months were included in the study. Six patients were lost to follow up and not included in the study; one patient had developed bronchogenic carcinoma and had expired; one individual developed sepsis necessitating implant removal; four patients developed worsening of the cuff tear necessitating a conversion to a reverse prosthesis. Of the 23 patients included in the study patients 21 had undergone hemi replacement and 2 had undergone total shoulder replacement arthroplasty (Fig. 2). The aetiology was idiopathic osteoarthritis (63.2%), congenital dysplasia (10.2%), osteonecrosis (10.5%), cuff arthropathy (5.3%) and post- traumatic arthritis (10.5%). One patient with bilateral shoulder dysplasia underwent a bilateral staged procedure for the same. The preoperative MRI demonstrated partial tear of the supraspinatus in 13 shoulders of which four had a fatty degeneration, partial tear of the infraspinatus in one shoulder, total rupture of the subscapularis in four and partial subscapularis tear in six shoulders. One patient had Nickel allergy and a gold-coated implant was used for the same.

Two patients required revision surgery, one in whom the glenoid base plate was changed from a metallic to polyethylene and the other patient had a periprosthetic fracture of the humerus.

Mean age at the time of surgery was 59.9 ± 9.2 yrs.; the mean duration of follow-up was 7.57 ± 1.09 yrs. The mean age adjusted Constant Score was 32.9 ± 5.2 pre- operative that improved to 78.9 ±20.1 at last follow up. The increase in the Constant Score was found to be statically significant between the pre-operative values and at the last follow up (*p* < 0.0001). The post op Subjective Shoulder Value (SSV) was measured to be 70.5 ± 21.1.

The change in the range of motion of the shoulder has been described in Table 1. There was a significant increase in the range of motion with respect to flexion, extension and external rotation between the pre-operative values and at the last follow up (p < 0.0001).

**Table 1:** Clinical assessment of the outcome

Duration	Flexion		Abduction		External rotation		CS	Age adjusted CS
	Active	Passive	Active	Passive	Active	Passive		
Pre-op	71.4 ± 15.9	90.9 ± 22.5	68.1 ± 13.2	86.6 ± 20	27.6 ± 11.9	33.1 ± 9.1	32.9 ±5.2	33.76 ± 5.1
72 mo	142.9 ± 36.6	160.5 ± 32.3	135.2 ± 40.5	148.5 ± 38.9	49.8 ± 21.9	51.6 ± 14.8	78.9 ±20.1	83 ± 15.9

*CS* Constant Score, *mo* months, *w* weeks, *y* years

Flexion/ Abduction and external rotation in degrees (°)

Serial AP and axillary lateral radiographs were done at follow up. The mean duration of the radiological follow up was 88.1 ±21.4 months. The system developed by Habermyer was used to classify the radiolucent lines < 2 mm [13] (Fig. 1). Radiolucent lines> 2 mm were observed in three shoulders. The presence of radiolucent lines < 2 mm did not influence the clinical outcome with regards to the post operative age adjusted CS, shoulder range of active or passive movement or the SSV (Table 2).

**Table 2:** Influence of radiological parameters on clinical outcome

Parameter	n		CS		*p* value	SSV		*p* value
	Absent	Present	Absent	Present		Present	Absent	
ap-a	19	3	43.7 ± 21.5	49.0 ± 21.6	0.5	90.0 ± 10	68.7 ± 21	0.1
ap-b	20	2	46.1 ± 21.6	45.0 ± 21.2	0.9	65.0 ± 49.5	73.2 ± 18.9	0.61
ap-c	19	3	46.8 ± 21.2	30.0	0	66.6 ± 32.1	73.4 ± 20	0.61
ax-a	20	2	46.6 ± 22.1	43.2 ± 17.7	0.78	85 ± 7	71.2 ± 21.7	0.39
ax-b	20	2	45.5 ± 22	49.0 ± 16.5	0.79	95 ± 7	70.2 ± 20.8	0.11
ax-c	18	4	43.4 ± 22.6	54.2 ± 13.7	0.3	75.0 ± 31.	71.9 ± 19.4	0.8
AP-A	12	10	43.2 ± 21.4	62.6 ± 4.9	0.1	74.5 ± 23.1	70.8 ± 20.2	0.69
AP-B	20	2	45.7 ± 21.3	48.0 ± 25.4	0.8	60.0 ± 42.4	73.7 ± 19.5	0.39
AP-C	21	1	45.22 ± 21.8	50.6 ± 18.3	0.6	30.0	74.5 ± 19.36	–
AX-A	18	4	44.4 ± 21.5	61 ± 5.6	0.3	70.0 ± 28.2	73.0 ± 20.2	0.8
AX-B	19	3	44.3 ± 21.4	61.5 ± 6.3	0.2	70.0 ± 34.6	72.8 ± 19.6	0.8
AX-C	17	5	44 ± 21.8	54.5 ± 16.8	0.3	75.0 ± 26.9	71.7 ± 20.0	0.7

ap a,b,c represent zones for measurement on the AP radiograph and ax a,b,c represent zones on the axillary radiograph

Small caps indicate bone density loss, Capitals indicate radiolucent lines

*n* number

*CS* Constant score

*SSV* Subjective shoulder value

Progressive cranial migration of the humeral head, with a decrease in acromio-humeral interval from 9.05 ± 3.6 mm to 5.01 ± 2.3 mm was observed at final follow up. Decrease in acromio-humeral interval correlated with poorer external rotation at follow up (Coefficient of correlation =1).

Secondary glenoid wear was seen in 10 shoulders, secondary osteophytes were seen in 7 shoulders. Radiolucent glenoid lines were not seen in any shoulders. The glenoid wear and secondary glenoid osteophytes had no significant bearing on the clinical outcome at the last follow up in terms of the measured parameters, age adjusted CS, SSV, post operative shoulder range both active and passive (Table 3).

**Table 3:** Influence of rotator cuff pathology on the clinical outcome

Outcome Measure	SST Partial tear			IST Partial tear			SSc tears				SST FD		
	No Tear(7)	PSST(21)	*p*-Value	NO tear (21)	PIST(1)	*P* val	No tear (10)	PSSC(7)	CT(4)	*P* value	Nil(17)	FD	*P* value
SSV	88.5 ± 12.1	65.3 ± 21.6	0.01	72 ± 21.4	95	–	82 ± 17.5	57.1 ± 23.6	78.7 ± 11.8	0.04	75.5 ± 21.9	62.5 ± 18.9	0.28
CS Increase	57.1 ± 17.2	40.43 ± 21	0.08	45.2 ± 21.3	61	–	51.5 ± 17.06	29.2 ± 22.2	61.5 ± 3.2	0.01	49.4 ± 21	31.2 ± 15.2	0.1
FE Increase(°)	84.2 ± 43.9	65 ± 36.9	0.3	70.5 ± 40.1	90	–	79 ± 39.5	54.2 ± 46.8	82.5 ± 15	0.3	74.1 ± 41.4	60 ± 28.2	0.5
Abduction Increase(°)	87.1 ± 23.6	57.1 ± 46.6	0.1	65.5 ± 42.7	100	–	75 ± 30.6	40 ± 55	95 ± 10	0.07	73.5 ± 43.9	40 ± 27	0.1
ER Increase(°)	35.7 ± 9.3	15.3 ± 30.6	0.1	21.2 ± 27.4	40	–	39 ± 14.3	7.8 ± 22.1	32.5 ± 9.5	0.00	20.2 ± 24.9	30 ± 38.3	0.5

*CS* Constant score, *FE* Forward Elevation, *ER* External rotation, *SST* Supraspinatus, *PSST* Partial tear of the supraspinatus, *IST* Infraspinatus, *PIST* Partial tear of the infraspinatus, *SSc* Subscapularis, *PSSC* Partial tear of the subscapularis, *CT* Complete tear of the subscapularis, *FD* Fatty degeneration

**Fig. 3:**
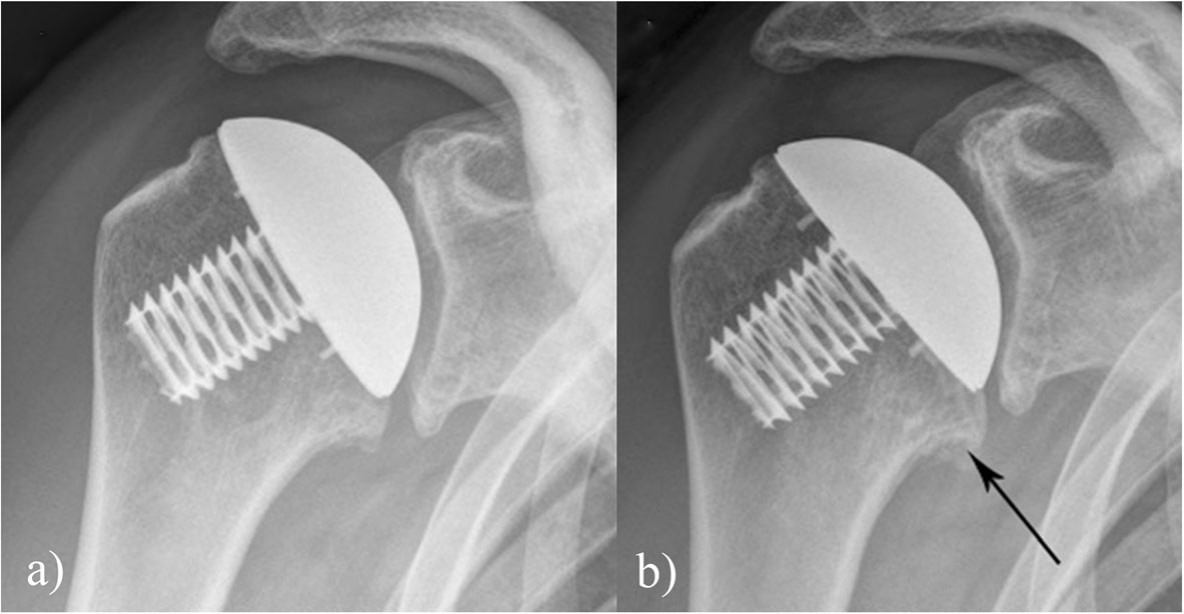
**a** Post operative image post surgery, **b** radiograph at eight years showing stress shielding around the calcar as indicated by the arrow

**Fig. 4:**
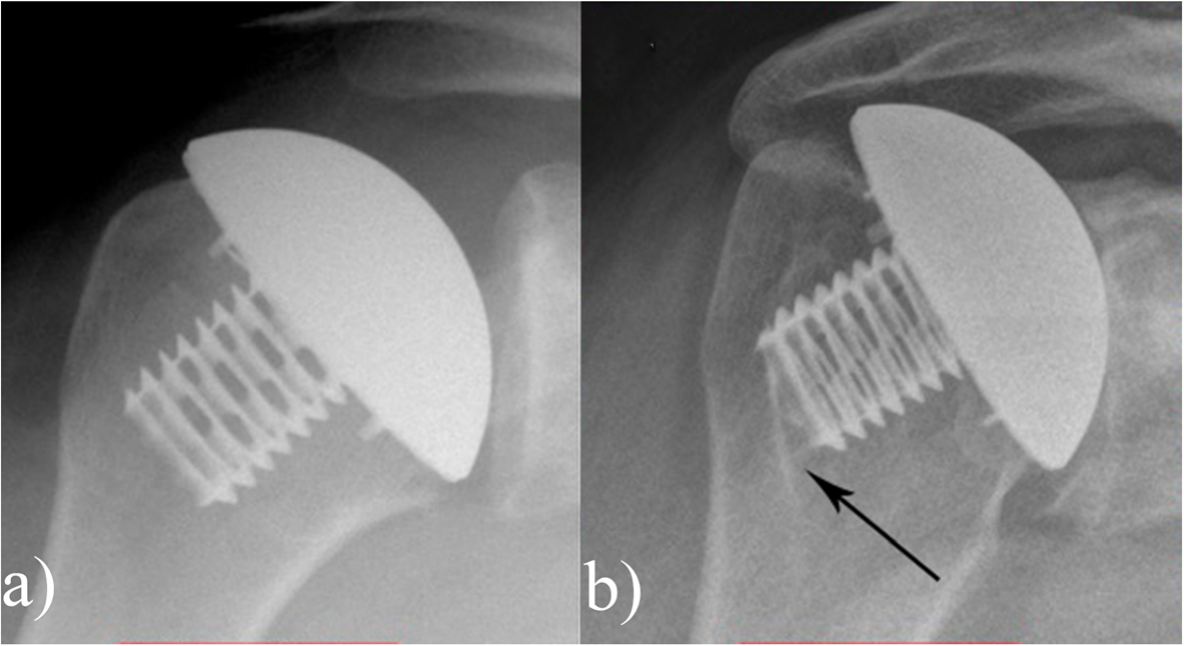
**a** Post operative image post surgery, **b** radiograph at six years showing stress shielding around the coring screw indicated by the arrow

There were two variations in the pattern of stress shielding observed: of the 13 shoulders with centred prosthesis 11 had evidence of bone on growth on the calcar (Fig. 3); three out of six patients with a decentred prosthesis showed evidence of stress shielding around the coring screw (Fig. 4). Clinical instability was not observed in any of the shoulders. On the glenoid aspect sclerosis was seen in 13 patients who had undergone hemi replacement.

The statistical assessment revealed that the presence of pre-operative partial supraspinatus tear led to inferior SSV (*p* = 0.01). Complete / partial tears of the subscapularis similarly had a negative impact on the outcome with worse SSV (*p* = 0.04), CS (0.01) and post- operative increase in external rotation (*p* < 0.5). However fatty infiltration of the supraspinatus, infraspinatus partial tears did not influence on the postoperative SSV, the age adjusted CS or the postoperative shoulder active /passive range (Table 4).

**Table 4:** Comparison of the present study to studies of conventional stemmed prosthesis

Study	No of shoulders	Duration of follow up	Prosthesis	Mean CS	Age adjusted CS	Range of motion (in degrees)					
						Flexion		Abduction		External rotation	
						A	P	A	P	A	P
Raiss et.al (2012)	46	10y	Aq	61 ± 18.2	79 ± 43	133 ± 36.7		123 ± 41.6		35 ± 15.8	
Deshmukh (2005)	72	14 y ± 2.7	N			Elevation-80 (58–100)	109 (84- 124)			25 (15–41)	
Levineet.al (2012)	27	17.2y(13–21)	N	–	–	Elevation- 141.89 (45-180)				61 (30–90)	
Sandow (2013)	33	3y	G	54.5 ± 4^a^ 77 ± 6^b^							
Favard (2012)	110	8y	Anatomic/ Reverse	62.8 o 47.5 r 53.8 c							
Orfaly et al. (2003)	65	4.3y(2–8)	G			Elevation 147				39	
Denard (2013)	50	115.5mo(60–211)	Aq	58.4 ± 20.7	73.4 ± 23.9	128 ± 36				33 ± 24	
Current study	21	5.9y(4.5–7.9)	E	71.2 ±16.6	83 ± 15.9	149.5 ± 38.9	160.4 ± 32.3	138 ± 43.4	148.5 ± 38.	46.1 ± 17.3	52.3 ± 14.3

Abbreviations, *y* year, *mo* months, ^a^hemiarthroplasty, ^b^total shoulder arthroplasty, *o* osteoarthritis, *r* heumatoid arthritis, *c* cuff arthropathy, *y* years, *mo* months, *E* Eclipse Prosthesis, *Aq* Aequalis prosthesis, *G* Global Arthroplasty system, *N* Neer II

Localised osteopenia was observed in the zones *a, b, c* in the anteroposterior and axillary radiographs. The presence of localised osteopenia did not influence the clinical outcome with regards to the measured clinical parameters (Table 2). Similar results were seen in other studies in this regard (Table 5).

**Table 5:** Comparison of the current study to resurfacing/ stemless implants

Study	No of shoulders	Follow up	Prosthesis	CS	Age adjusted CS	Range of motion					
						Flexion		Abduction		External rotation	
						Active	P	A	P	A	P
Thomas et.al (2005)	48	34.2 mo(24–63)	Co	48.6r 58o 54c		120 o 97 r		99 o 75 r		46 o 39 r	
Mullet (2007)	21	53.6 mo(25–111)	Co	56.4	77 (25–100)	105.9 (73.5 − 137.5)					
Levy et al. (2004)	62	6.5 yr(2–16)	Co	53.4 ± 13.8^a^ 47.9 ± 17.8^b^	71 ± 19.8^b^ 76 ± 13.4^a^	101 ± 41^b^ 104 ± 42^a^		83 ± 45 ^b^ 87 ± 43^a^		44 ± 24 ^b^ 47 ± 26^a^	
Berth et.al.(2013)	41	30.8 ± 4.6푚표	T	54.7 ± 7.3	73.2 ± 11.3	115.9 ± 9.8		105 ± 12.1		54.4 ± 10.7	
Razmjou et.al (2013)	17 t 39b	24 mo	T, B, N		92 ± 22 T	135 ± 35 T		121 ± 40 T		54 ± 17 t	
	18n				89 ± 18 퐵 94 ± 24 N	142 ± 24 B 131 ± 27 N		126 ± 33 B 123 ± 32 N		51 ± 18 b 43 ± 19 푛	
Huguet et.al (2010)	61	36 mo(36–51)	T	75		145				40	
Churchill et.al (2016)	149	2	S	80.7 ± 10.5	104.1 ± 14.8	146.6 ± 23.7		–	–	56.4 ± 15.4	
Current study	21	5.9 y(4.5–7.9)	E	71.2 ± 16.6	83 ± 15.9	149.5 ± 38.9	160.4 ± 32.3	138 ± 43.4	148.5 ± 38.5	46.1 ± 17.3	52.3 ±14.3
Habermyer (2015)	78	72.9 mo	E	75 ± 31	–	140.7 ± 35.9		129.9 ± 29.3		44.2 ± 20	
Hawi (2017)	43	9y(90-127mo)	E	79 ± 21	–	118 ± 43	–	105 ± 43		43 ± 19	
Gallacher(2018)	100	35.4 (24–76) mo	E	OSS: 28/48 (36-40)	–	–	–	–		–	
Uschok(2017)	14	68 (59–84) mo	E	72.8 ± 11	–	154 ± 8.5	–	149.3 ± 14.9		48.6 ± 15.6	

^a^Total shoulder arthroplasty. ^b^Hemiarthroplasty

*o* Osteoarthritis, *r* Rheumatoid arthritis, *c* Cuff arthropathy

*T* TESS: Total evolutive shoulder system

*B* Bigliani –Flatow

*N* Neer

*Co* Copeland (Mark III)

*S* Simpliciti shoulder prosthesis

*E* Eclipse shoulder prosthesis

## Discussion

In the current study, presence of radiolucent lines < 2 mm did not influence the clinical outcome with regards to the post operative age adjusted CS, shoulder range of active or passive movement or the SSV. Two patterns of stress shielding were observed, in centred prosthesis it was observed around the calcar in 11/13 shoulders while in decentred prosthesis it was seen around the coring screw in 3 out of 6 shoulders. Decrease in acromio-humeral distance on the radiographs led to a worsening outcome. Presence of pre-operative partial supraspinatus tear was a predictor for a poorer outcome with inferior SSV (*p* = 0.01). Complete / partial tears of the subscapularis similarly had a negative impact on the outcome with worse SSV (*p* = 0.04), CS (0.01) and post- operative increase in external rotation (*p* < 0.5).

Restoration of the proximal humeral anatomy involves accurate reproduction of the offset, version, the angulation of the head, the neck length and lastly the head diameter. The stemless Eclipse® prosthesis allows an accurate reproduction of these parameters; lack of a stem ensures replication of the offset, the neck cut allows accurate restoration of the angulation, the retroversion and the neck length [8].

The results of the current study are better compared to other studies done to assess the outcome of stemmed shoulder arthroplasty in terms of the Constant Score and the shoulder range of motion [4, 18–21] (Table 6) [4, 18–23]. However it must be mentioned that the duration of the follow up in our study was minimum 72 months, which was less than some of these studies. The clinical results of the stemless prosthesis are superior compared to the Copeland resurfacing. Additionally due to head resection the complications associated with the glenoid exposure in surface replacement are avoided [10] (Table 7) [5, 9, 10, 24–26].

**Table 6:** Comparison of the radiological parameters compared to other studies on the Eclipse prosthesis

Study	n	Radiolucent lines						Secondar y Glenoid wear	Glenoid RL < 1 mm	Bone density loss					
		ap -a	ap-b	ap-c	ax-a	ax-b	ax-c			ap -a	ap- b	ap- c	ax- a	ax- b	ax- c
Habermeyer (2015)	78	27%	7.9%	7.9%	22.2%	1.6%	1.6%	71.9%*	8.3%^a^ 53.3%^b^	41.3%					
Uschok (2017)	14	–	–	–	–	–	–	–	2	4	1	0	1	0	0
Gallacher (2018)	100	14	1	7	11	1	21	–	–	–	–	–	–	–	–
Hawi (2017)	43	2.3%						1	27.3%	29.4%					
Current Study	23	3	2	3	2	2	4	8	0	10	2	1	4	3	5

^a^Uncemented Metal back component

^b^All polythene glenoid

HA group

n = number of shoulders

ap-a,b,c represent zones in the anterioposterior radiographs

ax-a,b,c represent zones in axillary radiographs

**Table 7:** Radiological changes on the glenoid and effect on clinical outcome

Variable	Secondary Osteophytes		*P* value	Secondary Glenoid wear		*P*-Value
	Absent	Present		Absent	Present	
SSV	70.3 ± 22.7	77.4 ± 17.9	0.49	72.1 ± 20	73.1 ± 24.3	0.9
CS increase	44.3 ± 21.7	50.1 ± 20.4	0.57	47 ± 20.3	44.3 ± 23.4	0.7

*SSV* Subjective shoulder value, *CS* Constant Score

The results of the current study are similar in terms of the post -operative Constant Score and range of motion when compared to the other studies of stemless implants [5, 13, 27, 28] (Table 7).

Churchill et al. investigated the outcome of Simpliciti stemless prosthesis (Wright medical) in 157 patients with glenohumeral osteoarthritis. Of 157 patients 149 patients were available with a minimal follow up of 2 years. Range of motion increased from 103 ° to 147 ° and external rotation from 31 ° to 57 °. The authors described a subjective method of assessing the metaphyseal bone stock by compressing it with operating surgeons thumb; if there was no indentation the bone stock was considered adequate for implantation else a conventional prosthesis was used. The authors did not observe any component loosening, proximal migration of the humeral head [29].

Habermeyer et al. in a study of 78 patients with a minimal follow up of 72 months compared the results of Eclipse prosthesis in a osteoarthritis group (*n* = 39) to that of post traumatic arthritis group (*n* = 26). Both populations showed a significant increase in the CS and active range of motion post surgery. The osteoarthritis group had better increase in abduction compared to the posttraumatic arthritis group. The results of hemiarthroplasty compared to total shoulder arthroplasty were similar in both groups [13]. In our study the sample size of patients belonging to each aetiology group was less to derive a statistically significant correlation.

Uschok et.al in a randomised trial compared the outcomes of the Eclipse prosthesis to a conventional stemmed prosthesis and obtained similar results in terms of post operative Constant Score and improvement in range of movement over a period of the study of five years; they also observed that the positive results were maintained during the follow up. Also it was noted that the bone density was lowered in the calcar region (Zone c AP radiograph) in more individuals undergoing the conventional stemmed prosthesis compared to the Eclipse prosthesis [30].

Radiolucent lines were observed in zones *a, b, c* as described by Habermyer in the AP and axillary radiographs (Table 2, Fig. 1). Similarly localised osteopenia was observed on examination of the follow up radiographs; the results of which are summed up in Table 2 and comparison to other studies in this regard in Table 5. Golkhe et al. in a cadaveric study has suggested that radiolucent lines around stemless prosthesis may be a radiation artefact due to radiation scatter rather than true bone loss or stress shielding [31].

Stress shielding was another phenomenon observed during our study. The adaptation of the host bone in response to a prosthesis inserted in the medullary cavity is called stress shielding as per Wolff’s law [32]. Stress shielding can manifest as tuberosity resorption, cortical thinning, calcar osteolysis and occurs because of sharing of load between the prosthesis and bone; both material with different Young’s moduli [16]. The degree of stress shielding and the location varies depending on the prosthesis size and design [16]. Previous studies have indicated stress shielding around humeral resurfacing implants; especially around the rim and the core [33]. External stress shielding in form of calcar sclerosis was seen in the shoulders with a centred implant, whereas the internal stress shielding was seen in de-centred implant. Lesser bone density loss is seen in stemless prosthesis compared to stemmed prosthesis [30].

Edwards et.al in a multi centre study observed that supraspinatus tear in individuals undergoing shoulder arthroplasty had no influence on the post-op constant score or patient satisfaction. Repairing the supraspinatus tear had no influence on the clinical outcome other than an improvement in post operative external rotation; individuals with supraspinatus tears had lesser strength than individuals without the tear. Fatty infiltration of the infraspinatus, subscapularis had more negative impact on the outcome [14]. In contrast in our study we observed worse postoperative SSV in individuals having partial supraspinatus tear as well as tears of the subscapularis. Fatty infiltration of the supraspinatus had no influence on the final result.

Despite the good clinical outcomes and clinic-radiological outcomes similar to other studies if the stemless prosthesis, this study has a few drawbacks. The patient cohort was small to assess the effect of each of the pathologies leading to glenohumeral arthritis on the outcome. A hemiarthroplasty was done in a majority of the cases, however our main aim of the study was to assess the radiolucent lines that have been observed on the humeral side following a stemless shoulder arthroplasty. Additionally we lacked a control cohort to compare the clinical and radiological outcomes, as it was a retrospective study.

## Conclusions

An important message from the study is that localised osteopenia and radiolucent lines are common after stemless prosthesis implantation, and these are not predictors of worse clinical outcome in mid term; however it need to be seen in the longer run if these radiological findings assume any clinical significance. In presence of rotator cuff disease, stemless shoulder prosthesis needs to be approached with caution as inferior results are seen in presence of supraspinatus tears and subscapularis tears.

## Supplementary information

**Additional file 1.**

**Additional file 2.**

**Additional file 3.**

**Additional file 4.**

## Data Availability

All data generated or analysed during this study are included in this published article. The raw data is submitted as Additional files 1, 2, 3 and 4.

## Footnotes

1 Fiberwire® is a trademark of Arthrex Inc. Naples, Florida

## Abbreviations

CS: Constant score
L: Large
M: Medium
S: Small
SSV: Subjective shoulder value
