# Supplementary material for: Do the radiological changes seen at mid term follow up of stemless shoulder prosthesis affect outcome?
Source: BMC Musculoskelet Disord. 2019 Oct 27;20:490. doi: 10.1186/s12891-019-2870-z (PMC6815366; doi:10.1186/s12891-019-2870-z)
Supplement: Supplementary file 1 — Additional file 1. [file 12891_2019_2870_MOESM1_ESM.docx]

|  | Mean | Std. Dev. | Min | Max |
| --- | --- | --- | --- | --- |
|  |  |  |  |  |
| age | 59.91 | 9.26 | 44.00 | 75.00 |

|  | Mean | Std. Dev. | Min | Max |
| --- | --- | --- | --- | --- |
|  |  |  |  |  |
| interval | 7.57 | 1.09 | 4.96 | 9.66 |

|  | Mean | Std. Dev. | 95% Conf. Interval | | P value |
| --- | --- | --- | --- | --- | --- |
|  |  |  |  |  |  |
| actfle~2 | 142.86 | 36.63 | 126.19 | 159.53 |  |
| actfle~1 | 71.43 | 15.90 | 64.19 | 78.67 |  |
|  |  |  |  |  |  |
| diff | 71.43 | 39.41 | 53.49 | 89.37 | <0.0001 |

|  | Mean | Std. Dev. | 95% Conf. Interval | | P value |
| --- | --- | --- | --- | --- | --- |
|  |  |  |  |  |  |
| actabd~2 | 135.24 | 40.57 | 116.77 | 153.71 |  |
| actabd~1 | 68.10 | 13.27 | 62.05 | 74.14 |  |
|  |  |  |  |  |  |
| diff | 67.14 | 42.33 | 47.88 | 86.41 | <0.0001 |

|  | Mean | Std. Dev. | 95% Conf. Interval | | P value |
| --- | --- | --- | --- | --- | --- |
|  |  |  |  |  |  |
| actaro2 | 49.76 | 21.99 | 39.75 | 59.77 |  |
| actaro1 | 27.62 | 11.90 | 22.20 | 33.03 |  |
|  |  |  |  |  |  |
| diff | 22.14 | 27.04 | 9.83 | 34.45 | 0.0013 |

|  | Mean | Std. Dev. | 95% Conf. Interval | | P value |
| --- | --- | --- | --- | --- | --- |
|  |  |  |  |  |  |
| pasfle~2 | 160.48 | 32.32 | 145.76 | 175.19 |  |
| pasfle~1 | 90.95 | 22.56 | 80.68 | 101.22 |  |
|  |  |  |  |  |  |
| diff | 69.52 | 43.64 | 49.66 | 89.39 | <0.0001 |

|  | Mean | Std. Dev. | 95% Conf. Interval | | P value |
| --- | --- | --- | --- | --- | --- |
|  |  |  |  |  |  |
| pasabd~2 | 148.57 | 38.90 | 130.87 | 166.28 |  |
| pasabd~1 | 86.67 | 20.08 | 77.52 | 95.81 |  |
|  |  |  |  |  |  |
| diff | 61.90 | 48.33 | 39.90 | 83.91 | <0.0001 |

|  | Mean | Std. Dev. | 95% Conf. Interval | | P value |
| --- | --- | --- | --- | --- | --- |
|  |  |  |  |  |  |
| cs2 | 78.90 | 20.19 | 69.71 | 88.09 |  |
| cs1 | 32.90 | 5.23 | 30.52 | 35.29 |  |
|  |  |  |  |  |  |
| diff | 46.00 | 21.04 | 36.42 | 55.58 | <0.0001 |

|  | Mean | Std. Dev. | Min | Max |
| --- | --- | --- | --- | --- |
|  |  |  |  |  |
| ssv | 72.50 | 21.14 | 30.00 | 100 |

|  | cranini | cranfu | diffac~n | diffac~t | diffac~o | diffpa~n | diffpa~t | diffpa~o | diffcs | ssv |
| --- | --- | --- | --- | --- | --- | --- | --- | --- | --- | --- |
|  |  |  |  |  |  |  |  |  |  |  |
| cranini | 1.00 |  |  |  |  |  |  |  |  |  |
|  |  |  |  |  |  |  |  |  |  |  |
|  |  |  |  |  |  |  |  |  |  |  |
| cranfu | 0.53 | 1.00 |  |  |  |  |  |  |  |  |
| P value | 0.01 |  |  |  |  |  |  |  |  |  |
|  |  |  |  |  |  |  |  |  |  |  |
| diffactfle~n | 0.19 | 0.16 | 1.00 |  |  |  |  |  |  |  |
| P value | 0.42 | 0.48 |  |  |  |  |  |  |  |  |
|  |  |  |  |  |  |  |  |  |  |  |
| diffactabd~t | 0.06 | 0.23 | 0.76 | 1.00 |  |  |  |  |  |  |
| P value | 0.78 | 0.31 | <0.01 |  |  |  |  |  |  |  |
|  |  |  |  |  |  |  |  |  |  |  |
| diffactaro | 0.42 | 0.64 | 0.22 | 0.26 | 1.00 |  |  |  |  |  |
| P value | 0.06 | <0.01 | 0.34 | 0.25 |  |  |  |  |  |  |
|  |  |  |  |  |  |  |  |  |  |  |
| diffpasfle~n | 0.00 | -0.01 | 0.76 | 0.68 | 0.13 | 1.00 |  |  |  |  |
| P value | 1.00 | 0.96 | <0.01 | <0.01 | 0.56 |  |  |  |  |  |
|  |  |  |  |  |  |  |  |  |  |  |
| diffpasabd~t | 0.00 | 0.12 | 0.69 | 0.87 | 0.25 | 0.78 | 1.00 |  |  |  |
| P value | 1.00 | 0.61 | <0.01 | <0.01 | 0.27 | <0.01 |  |  |  |  |
|  |  |  |  |  |  |  |  |  |  |  |
| diffpasaro | 0.05 | 0.20 | 0.38 | 0.40 | 0.73 | 0.43 | 0.49 | 1.00 |  |  |
| P value | 0.84 | 0.39 | 0.09 | 0.08 | <0.01 | 0.05 | 0.02 |  |  |  |
|  |  |  |  |  |  |  |  |  |  |  |
| diffcs | 0.30 | 0.55 | 0.78 | 0.81 | 0.48 | 0.56 | 0.73 | 0.49 | 1.00 |  |
| P value | 0.19 | 0.01 | <0.01 | <0.01 | 0.03 | 0.01 | <0.01 | 0.02 |  |  |
|  |  |  |  |  |  |  |  |  |  |  |
| ssv | 0.07 | 0.35 | 0.41 | 0.59 | 0.54 | 0.35 | 0.50 | 0.67 | 0.72 | 1.00 |
| P value | 0.77 | 0.11 | 0.06 | <0.01 | 0.01 | 0.12 | 0.02 | <0.01 | <0.01 |  |
