# Supplementary material for: Do the radiological changes seen at mid term follow up of stemless shoulder prosthesis affect outcome?
Source: BMC Musculoskelet Disord. 2019 Oct 27;20:490. doi: 10.1186/s12891-019-2870-z (PMC6815366; doi:10.1186/s12891-019-2870-z)
Supplement: Supplementary file 2 — Additional file 2. [file 12891_2019_2870_MOESM2_ESM.docx]

| SSPCT |  |  |  |  |  |
| --- | --- | --- | --- | --- | --- |
|  |  |  |  |  |  |
|  | No tear (7) | | Partial tear (14) | | P value |
| Variable | Mean | Std. Dev. | Mean | Std. Dev. |  |
|  |  |  |  |  |  |
| ssv | 88.57 | 12.15 | 65.36 | 21.16 | 0.015 |
| diffcs | 57.14 | 17.25 | 40.43 | 21.06 | 0.086 |
| diffactfle~n | 84.29 | 43.92 | 65.00 | 36.95 | 0.302 |
| diffactabd~t | 87.14 | 23.60 | 57.14 | 46.65 | 0.129 |
| diffactaro | 35.71 | 9.32 | 15.36 | 30.60 | 0.105 |
| diffpasfle~n | 84.29 | 51.92 | 62.14 | 38.86 | 0.284 |
| diffpasabd~t | 72.86 | 38.61 | 56.43 | 53.00 | 0.477 |
| diffpasaro | 24.29 | 9.76 | 15.00 | 19.01 | 0.243 |

| ISPCT |  |  |  |  |  |
| --- | --- | --- | --- | --- | --- |
|  |  |  |  |  |  |
|  | No tear (21) | | Partial tear (1) | |  |
| Variable | Mean | Std. Dev. | Mean | Std. Dev | P value |
|  |  |  |  |  |  |
| ssv | 72.00 | 21.42 | 95.00 |  |  |
| diffcs | 45.25 | 21.30 | 61.00 |  |  |
| diffactfle~n | 70.50 | 40.19 | 90.00 |  |  |
| diffactabd~t | 65.50 | 42.73 | 100.00 |  |  |
| diffactaro | 21.25 | 27.43 | 40.00 |  |  |
|  |  |  |  |  |  |
| diffpasfle~n | 70.00 | 44.72 | 60.00 |  |  |
| diffpasabd~t | 62.00 | 49.59 | 60.00 |  |  |
| diffpasaro | 17.50 | 17.05 | 30.00 |  |  |

| SCPCT |  |  |  |  |  |  |  |
| --- | --- | --- | --- | --- | --- | --- | --- |
|  |  |  |  |  |  |  |  |
|  | No tear (10) | | Partial tear (7) | | Complete tear (4) | | P value |
| Variable | Mean | Std. Dev. | Mean | Std. Dev. | Mean | Std. Dev. |  |
|  |  |  |  |  |  |  |  |
| ssv | 82.00 | 17.51 | 57.14 | 23.60 | 78.75 | 11.81 | 0.044 |
| diffcs | 51.50 | 17.06 | 29.29 | 22.28 | 61.50 | 3.42 | 0.017 |
| diffactfle~n | 79.00 | 39.57 | 54.29 | 46.85 | 82.50 | 15.00 | 0.386 |
| diffactabd~t | 75.00 | 30.64 | 40.00 | 55.08 | 95.00 | 10.00 | 0.077 |
| diffactaro | 39.00 | 14.30 | -7.86 | 22.15 | 32.50 | 9.57 | 0.000 |
| diffpasfle~n | 81.00 | 43.06 | 54.29 | 51.27 | 67.50 | 29.86 | 0.483 |
| diffpasabd~t | 71.00 | 33.81 | 30.00 | 61.10 | 95.00 | 23.80 | 0.063 |
| diffpasaro | 27.00 | 11.60 | 1.43 | 12.82 | 25.00 | 13.54 | 0.002 |

| SSPFD |  |  |  |  |  |
| --- | --- | --- | --- | --- | --- |
|  |  |  |  |  |  |
|  | No degeneration (17) | | Fatty degeneration (4) | | P value |
| Variable | Mean | Std. Dev. | Mean | Std. Dev. |  |
|  |  |  |  |  |  |
| ssv | 75.59 | 21.79 | 62.50 | 18.93 | 0.284 |
| diffcs | 49.47 | 21.04 | 31.25 | 15.22 | 0.122 |
| diffactfle~n | 74.12 | 41.84 | 60.00 | 28.28 | 0.533 |
| diffactabd~t | 73.53 | 43.29 | 40.00 | 27.08 | 0.159 |
| diffactaro | 20.29 | 24.90 | 30.00 | 38.30 | 0.532 |
| diffpasfle~n | 72.35 | 46.17 | 57.50 | 33.04 | 0.554 |
| diffpasabd~t | 64.71 | 50.88 | 50.00 | 39.16 | 0.597 |
| diffpasaro | 16.18 | 16.35 | 26.25 | 18.87 | 0.293 |

| APA BD |  |  |  |  |  |
| --- | --- | --- | --- | --- | --- |
|  | Absent (12) | | Present (10) | | P value |
| Variable | Mean | Std. Dev. | Mean | Std. Dev. |  |
|  |  |  |  |  |  |
| ssv | 70.83 | 20.21 | 74.50 | 23.15 | 0.696 |
| diffcs | 43.75 | 21.52 | 49.00 | 21.26 | 0.585 |

| ABB BD |  |  |  |  |  |
| --- | --- | --- | --- | --- | --- |
|  | Absent (20) | | Present (2) | | P value |
| Variable | Mean | Std. Dev. | Mean | Std. Dev. |  |
|  |  |  |  |  |  |
| ssv | 73.75 | 19.53 | 60.00 | 42.43 | 0.394 |
| diffcs | 46.11 | 21.60 | 45.00 | 21.21 | 0.946 |

| APC BD |  |  |  |  |  |
| --- | --- | --- | --- | --- | --- |
|  | Absent (21) | | Present (1) | |  |
| Variable | Mean | Std. Dev. | Mean | Std. Dev. |  |
|  |  |  |  |  |  |
| ssv | 74.52 | 19.36 | 30.00 | . |  |
| diffcs | 46.80 | 21.26 | 30.00 | . |  |

| AXA BD |  |  |  |  |  |
| --- | --- | --- | --- | --- | --- |
|  | Absent (18) | | Present (4) | | P value |
| Variable | Mean | Std. Dev. | Mean | Std. Dev. |  |
|  |  |  |  |  |  |
| ssv | 73.06 | 20.23 | 70.00 | 28.28 | 0.801 |
| diffcs | 46.65 | 22.18 | 43.25 | 17.73 | 0.780 |

| AXB BD |  |  |  |  |  |
| --- | --- | --- | --- | --- | --- |
|  | Absent (19) | | Present (3) | | P value |
| Variable | Mean | Std. Dev. | Mean | Std. Dev. |  |
|  |  |  |  |  |  |
| ssv | 72.89 | 19.67 | 70.00 | 34.64 | 0.832 |
| diffcs | 45.50 | 22.06 | 49.00 | 16.52 | 0.797 |

| AXC BD |  |  |  |  |  |
| --- | --- | --- | --- | --- | --- |
|  | Absent (17) | | Present (5) | | P value |
| Variable | Mean | Std. Dev. | Mean | Std. Dev. |  |
|  |  |  |  |  |  |
| ssv | 71.76 | 20.07 | 75.00 | 26.93 | 0.772 |
| diffcs | 43.44 | 22.60 | 54.20 | 13.70 | 0.331 |

| Sec glenoid | |  |  |  |  |
| --- | --- | --- | --- | --- | --- |
|  | Absent (14) | | Present (8) | | P value |
| Variable | Mean | Std. Dev. | Mean | Std. Dev. |  |
|  |  |  |  |  |  |
| ssv | 72.14 | 20.07 | 73.13 | 24.34 | 0.920 |
| diffcs | 47.00 | 20.34 | 44.38 | 23.47 | 0.789 |

| Sec Osteo | |  |  |  |  |
| --- | --- | --- | --- | --- | --- |
|  | Absent (15) | | Present (7) | | P value |
| Variable | Mean | Std. Dev. | Mean | Std. Dev. |  |
|  |  |  |  |  |  |
| ssv | 70.33 | 22.71 | 77.14 | 17.99 | 0.495 |
| diffcs | 44.33 | 21.73 | 50.17 | 20.47 | 0.579 |
