# Supplementary material for: Do the radiological changes seen at mid term follow up of stemless shoulder prosthesis affect outcome?
Source: BMC Musculoskelet Disord. 2019 Oct 27;20:490. doi: 10.1186/s12891-019-2870-z (PMC6815366; doi:10.1186/s12891-019-2870-z)
Supplement: Supplementary file 3 — Additional file 3. [file 12891_2019_2870_MOESM3_ESM.docx]

| APA RL |  |  |  |  |  |
| --- | --- | --- | --- | --- | --- |
|  | No Line (19) | | Line Present (3) | | P value |
| ssv | 69.74 | 21.24 | 90.00 | 10.00 | 0.126 |
| diffcs | 43.22 | 21.47 | 62.67 | 4.93 | 0.142 |

| APB RL |  |  |  |  |  |
| --- | --- | --- | --- | --- | --- |
|  | No Line (20) | | Line Present (2) | | P value |
| ssv | 73.25 | 18.94 | 65.00 | 49.50 | 0.611 |
| diffcs | 45.79 | 21.34 | 48.00 | 25.46 | 0.892 |

| APC RL |  |  |  |  |  |
| --- | --- | --- | --- | --- | --- |
|  | No Line (19) | | Line Present (3) | | P value |
| ssv | 73.42 | 20.00 | 66.67 | 32.15 | 0.619 |
| diffcs | 45.22 | 21.84 | 50.67 | 18.34 | 0.689 |

| AXA RL |  |  |  |  |  |
| --- | --- | --- | --- | --- | --- |
|  | No Line (20) | | Line Present (2) | | P value |
| ssv | 71.25 | 21.76 | 85.00 | 7.07 | 0.394 |
| diffcs | 44.42 | 21.51 | 61.00 | 5.66 | 0.301 |

| AXB RL |  |  |  |  |  |
| --- | --- | --- | --- | --- | --- |
|  | No Line (20) | | Line Present (2) | | P value |
| ssv | 70.25 | 20.80 | 95.00 | 7.07 | 0.117 |
| diffcs | 44.37 | 21.45 | 61.50 | 6.36 | 0.285 |

| AXC RL |  |  |  |  |  |
| --- | --- | --- | --- | --- | --- |
|  | No Line (18) | | Line Present (4) | | P value |
| ssv | 71.94 | 19.49 | 75.00 | 31.09 | 0.801 |
| diffcs | 44.00 | 21.86 | 54.50 | 16.82 | 0.383 |
